# Exploring Self-Reported Physical Activity Levels and Physical Fitness in Italian Children: A Mediation and Moderation Analysis

**DOI:** 10.3390/children12020204

**Published:** 2025-02-08

**Authors:** Domenico Monacis, Italo Sannicandro, Dario Colella

**Affiliations:** 1 Department of Education and Sport Sciences, Pegaso Telematic University, 80143 Naples, Italy; 2 Department of Clinical and Experimental Medicine, University of Foggia, 71122 Foggia, Italy; italo.sannicandro@unifg.it; 3 Department of Biological and Environmental Sciences and Technologies, University of Salento, 73100 Lecce, Italy; dario.colella@unisalento.it

**Keywords:** regional observatory of motor assessment, physical fitness, obesity, sedentary behavior, daily physical activity

## Abstract

Background/Objectives: Skill-related physical fitness is a crucial factor influencing health status during childhood. Starting from the lack of knowledge in the field of skill-related physical fitness and levels of physical activity in Southern Italy, this cross-sectional study aims to explore the mediating and moderating influence of PAL on the association between BMI and skill-related physical fitness. Methods: The sample (N = 387 students; male = 196, age = 12.2 ± 0.87 yrs; female = 191, age = 12.3 ± 0.93) was recruited from schools in the Apulia Region. The assessment included standing long jump (SLJ), 2 kg medicine ball throw (MBT) and 10 × 5 shuttle run (10 × 5) as indicators of physical fitness, and a self-report for measuring daily physical activity (PAL). A 2 (gender) × 3 (BMI cutoff)-factorial ANOVA was performed to highlight differences between groups for physical fitness components and PAL. Then, mediation and moderation models were created, establishing BMI as the independent variable, physical fitness tests as dependent variables, and PAL as the mediator and moderator variable. Results: The findings showed higher physical fitness and PAL in male and female normal-weight groups than in those who were overweight and obese. Mediation analysis revealed that higher PAL improved performance in SLJ (b = 0.091, *p* < 0.01, R^2^ = 17.16%), led to a modest understanding of the variation in MBT (ΔR^2^ = 0.026), and significantly reduced the time spent to perform 10 × 5 (*p* < 0.0, R^2^ = 10.72%). Conclusions: Despite the obtained results, future research is needed to further understand the association between physical fitness and the sociocultural determinants of physical activity to guide institutional policies and interventions to prevent poor health habits.

## 1. Introduction

The advantages of physical activity and healthy lifestyles during the lifespan have been widely documented in international public health research [1,2]. The enhanced functioning of the cardiovascular, respiratory, and immune systems [3] and improved brain development and synaptogenesis [4,5], muscle and bone health, body weight management, and metabolism [6,7] are among the main benefits. Additionally, active lifestyles and healthy behaviors throughout life best contribute to a significant decrease in the risk of cardiovascular and metabolic diseases, and type-2 diabetes [8]. Moreover, they also lower the risk of several types of cancer (e.g., colon, stomach, lung, bladder, etc.) [9], as well as the onset of anxiety, stress, and depression [10].

However, despite the WHO guidelines [11] recommending that children and adolescents should be engaged in at least 60 min of moderate to vigorous daily physical activity (MVPA), recent studies indicate that boys and girls between 11 and 17 years old do not engage in sufficient physical activity daily [12,13]. The increasing adoption of sedentary lifestyles and sitting activities is among the main reasons for what has been termed as one of the major public health issues of the 21st century [12,13,14,15].

Epidemiological data are even more worrying when considered in view of the effects of the recent COVID-19 pandemic. The restrictions implemented during quarantine have resulted in an even greater reduction in opportunities for physical activity [16,17,18] and the rise in psychological diseases (e.g., anxiety, depression, and eating disorders, etc.) among young people [19,20]. These negative trends, besides being significant for the rise in the percentage of overweight and obesity in children and adolescents, are among the main determinants of the decline in levels of physical activity as well as motor skill development and coordination [21].

This negative trend results in restricted opportunities for the development of physical fitness, defined as a positive state of well-being influenced by regular physical activity, genetic makeup, and nutritional adequacy, which includes aerobic endurance, muscular strength, muscular endurance, joint flexibility, and body composition [22,23]. Additionally, physical fitness is categorized into two main components: health-related physical fitness, which encompasses muscular strength and endurance, cardiovascular endurance, flexibility, and body composition, and skill-related physical fitness, which includes agility, coordination, power, speed, and balance [22,23,24,25].

During the last few decades, the association between physical fitness and health effects has emerged in international scientific debate, alongside attitudes and changes toward physical activity, becoming a significant indicator of health status from childhood to adolescence [26,27]. Thus, the global decline in physical fitness and rising pandemic of overweight and obesity can be interpreted in the light of unhealthy habits and poor lifestyles defined as activities with an energy expenditure of 1.5 METs (metabolic equivalent tasks) or lower (e.g., increased screen time, addiction to smartphones and social media, unbalanced diet, increased automobile travel, sitting time, etc.) [11], which are significant predictors of sedentary behavior and poor health in adulthood [28,29]. Despite the fact that higher fitness and levels of physical activity (PALs) lead to the minimization of negative consequences associated with high body mass index and contribute to positive effects on health [30], recent international monitoring and surveillance of physical activity reported that approximately 32% to 8–9-year-old Italian children achieved the sufficient physical activity levels recommended by the WHO [11]. These epidemiological data highlight the concerning and alarming reduction in the percentage of children who are physically active: in the eleven-year-old age group, only 11.9% engaged in sufficient daily physical activity, while among thirteen- and fifteen-year-olds, the percentages are approximately 9.3% and 6.8%, respectively [31]. Cross-sectional research studies carried out on Italian children suggest that reduced daily physical activity and higher BMI have a negative impact on children’s physical fitness and overall health status [32,33].

Moreover, recent findings showed an international and widespread inverse dose–response association between the improvement of cardiorespiratory fitness and all causes of mortality, including cardiovascular disease and cancer, independent of gender, age, and race [34,35]. A study conducted on 378 Portuguese children aged 9–11 years highlighted the important role of muscle strength in attenuating the effects of physical inactivity on metabolic risk: children classified as inactive but stronger had lower metabolic risk than inactive and “low-strength” peers [36]. In fact, lower levels of physical activity and physical fitness are associated with overweight and obesity, as an expression of reduced levels of motor skill development. Moreover, findings revealed that this unhealthy association is stronger in boys than in girls [37,38,39].

Despite this well-known association between health-related physical fitness and PAL, there is a lack of knowledge in the field of skill-related physical fitness and PAL in children in Southern Italy.

The literature analysis posed the following research questions:

(R1) Do skill-related physical fitness and PAL differ according to gender and BMI cutoffs in Italian children?

(R2) Does PAL mediate and moderate the relationship between BMI and skill-related physical fitness components? Which components of physical fitness can be best influenced by PAL?

To test the hypothesis that BMI may contribute to explaining variance in physical fitness, and that this association could be indirect via PAL, mediation models were created. Furthermore, moderation models were developed to determine whether the association between BMI and physical fitness was dependent on PAL.

## 2. Materials and Methods

### 2.1. Participants

This study was supported by the University of Salento (Lecce, Apulia). The study population was composed of Italian secondary schoolchildren, with no disabilities or mental/physical impairments or disorders. Participants were recruited from schools that joined the ROMA Project (Regional Observatory of Motor Assessment) in the Apulia Region of Southern Italy. The sampling strategy was based on the choice of the authors to control for specific variables that could add significant confounding factors to the study, as highlighted in international literature on the topic [40,41]

Before starting this study, a priori power calculations were conducted with G*Power [42,43] to identify the minimum number of participants required to achieve sufficient power (α = 0.05; expected power = 0.95). This study utilized a cross-sectional design. A multistage sampling procedure was applied to recruit participants (N = 400 students; male = 200, female = 200) from six Apulian provinces (Foggia, BAT, Bari, Lecce, Brindisi, and Taranto) to enhance the representativeness in the Apulia target population. However, the total sample consisted of 387 children aged 11–14 years (male = 196; female = 191), as 13 children did not attend all assessment procedures. The Committee established by the University of Salento regarding data collection on humans concerns psychological-medical research. All other types of research do not include an Ethics Committee. Informed consent was obtained from all children involved in the project before data collection.

### 2.2. Assessment Procedure

After recording weight and height, participants were classified as normal weight, overweight, or obese according to Cole’s Scale [44]. For the physical fitness assessment, lower- and upper-limb muscular strength were evaluated with standing long jump (SLJ) and 2 Kg medicine ball throw (MBT) tests, respectively [45,46], while speed and agility were assessed through the 10 × 5 m shuttle run test [47]. These physical fitness tests are reliable and field-based assessments for evaluating lower- and upper-limb explosive strength, and speed–agility [48], for skill-related fitness assessment as in previous studies in the Apulia Region [49]. Furthermore, data on these components of physical fitness are indirectly linked with middle-to-low motor development during adolescence [45].

Additionally, children were asked to complete the Italian version of the Physical Activity Questionnaire for Older Children [50,51,52,53,54] to evaluate self-reported daily physical activity practice over the last week. Physical education teachers in the Apulia Region and graduates in Sports Sciences were initially recruited in the ROMA Project and trained on the assessment procedure. Subsequently, they conducted the evaluations from October to December 2023. Assessments were carried out only in the schools that participated in the ROMA Project during curricular physical education lessons.

### 2.3. Statistical Analysis

After testing normality with the Kolmogorov–Smirnov test (sample size > 50), descriptive statistics for all anthropometric data, physical fitness tests, and levels of physical activity were conducted out in terms of mean, standard deviation, minimum, and maximum. Furthermore, a 2 (gender) × 3 (BMI cutoff)-factorial ANOVA was performed to highlight differences between groups for physical fitness components and levels of physical activity. Levene’s test was used to assess the equality of variance assumption. Both the main effect and interaction effect of gender (male/female) and BMI cutoff (normal weight/overweight/obese) on physical fitness tests and levels of physical activity were carried out. Since group sizes were unequal, Gabriel’s procedure was applied to conduct post hoc analysis. Moreover, partial eta squared (ηp2) and Cohen’s d were calculated as measures of effect size as follows: ηp2 = 0.01 (small effect); ηp2 = 0.06 (small effect), and ηp2 = 0.14 (large effect); Cohen’s d = 0.2 for small effect, Cohen’s d = 0.5 for medium effect, and Cohen’s d = 0.8 for large effect [55]

Then, Pearson’s r coefficient was used to highlight significant relationships between variables; the results were interpreted as follows: small correlation < 0.30; 0.30 < medium correlation < 0.50; and large correlation > 0.50.

After verifying the relationship between variables, the mediation analysis was applied to assess if physical activity levels (mediation variable) mediate the relationship between BMI, as the independent variable, and physical fitness tests as the dependent variable. The analysis was conducted according to the Preacher and Hayes methods [56]. Results were interpreted as follows: mediation was defined as partial if the indirect effect was significant, while it was defined as total if the indirect effect was non-significant. Significance was verified with the Bootstrapping method of Preacher and Hayes [55,56].

Moreover, according to Hayes (2018), the moderation analysis was carried out to study the interaction effect between BMI and PAL to explain the direction or magnitude of the relationship between BMI and physical fitness components, after adding the moderation variable [57]. All significant indices were set at *p*-value < 0.05. SPSS software vers. 26.0 for Windows and the PROCESS macro for SPSS were used for data analysis.

## 3. Results

The results of descriptive statistics have been reported in Table 1 according to gender and BMI cutoff. Due to missing values (male = 4, female = 9) in anthropometric characteristics and the physical fitness assessment, the final sample consists of 387 children. The main results of the descriptive analysis reveal better physical fitness indicators and PAL for both male and female normal-weight groups compared to overweight and obese ones.

Moreover, the F value for gender has a significant main effect on SLJ (F = 38.604, *p* < 0.01), MBT (F = 8.525, *p* < 0.01), and 10 × 5 (F = 32.736, *p* < 0.01), but not on PAL, while BMI cutoff significantly affects SLJ (F = 24.824, *p* < 0,01), MBT (F = 7.363, *p* < 0.01), 10 × 5 (F = 22.234, *p* < 0.01), and PAL (F = 8.229, *p* < 0.01), as can be seen in Table 2. No significant interaction effects between factors were found, except for PAL (F = 3.452, *p* < 0.01).

Since the main effects of gender and cutoff are almost all significant, post hoc analysis was performed to determine the simple effects of each variable (see Table 3).

The results highlighted that boys performed significantly better in all physical fitness tests and were more physically active than girls. Moreover, Nw showed overall significantly higher physical fitness and PAL compared to Ow and Ob in all tests, except for MBT.

In the male sample, ANOVA analysis revealed significant differences in SLJ (F = 16.771, *p* < 0.01; ηp^2^ = 0.148) and PAL (F = 11.850, *p* < 0.01; ηp^2^ = 0.129) with large effect sizes according to BMI cutoff. A significant effect was also reported for 10 × 5 (F = 7.682, *p* < 0.01) with a medium effect size (ηp^2^ = 0.074). In females, BMI cutoff significantly influenced SLJ (F = 9.150, *p* < 0.01; ηp^2^ = 0.089), MBT (F = 9.827, *p* < 0.01; ηp^2^ = 0.095), and 10 × 5 (F = 14.231, *p* < 0.01; ηp^2^ = 0.131). No significant differences were highlighted for MBT in males and PAL in females.

The correlation matrix between BMI and other variables is reported in Table 4. The results show an inverse significant association between BMI, SLJ (r = −0.257, *p* < 0.01), and PAL (r = −0.228, *p* < 0.01). At the same time, increasing BMI increases motor performance in MBT (r = 0.289, *p* < 0.01) and the time required to perform 10 × 5 (r = 0.224, *p* < 0.01). Subsequently, the mediation analysis was performed based on the correlation matrix.

**Figure 1:**
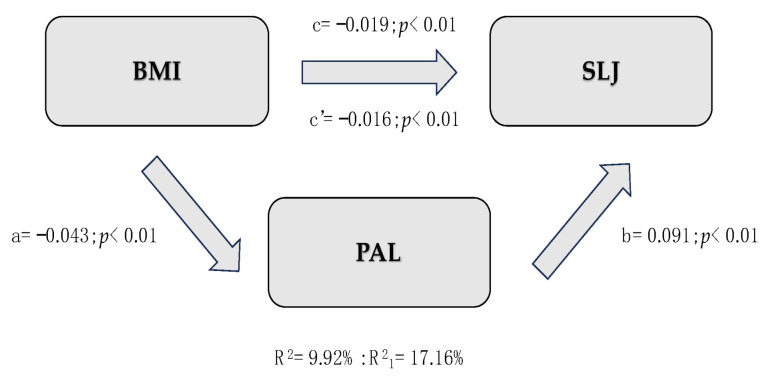
Representation of Mediation Model 1.

**Figure 2:**
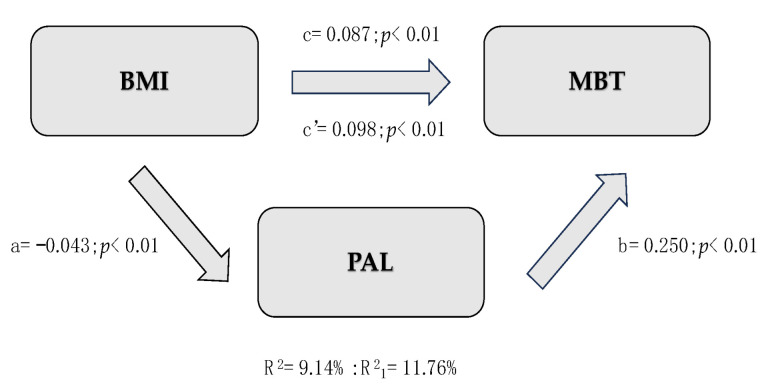
Representation of Mediation Model 2.

**Figure 3:**
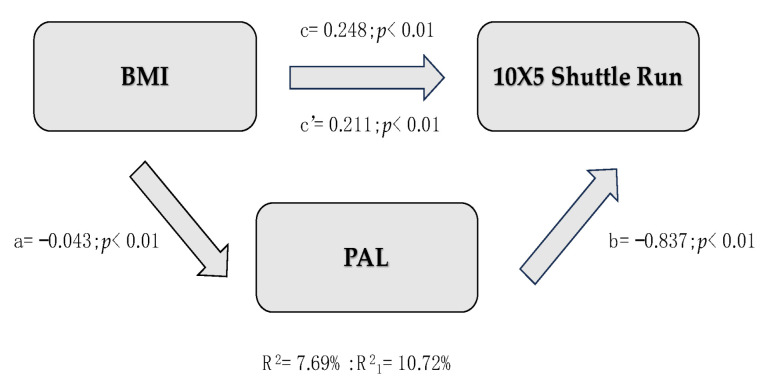
Representation of Mediation Model 3.

Table 5 provides a synthetic description of the three mediation models created. All models assume BMI as the IV, PAL as the MV, and physical fitness tests (SLJ, MBT, and *10 × 5*) as DVs. Since all indirect effects were significant, PAL is a partial mediator between variables. In the first model (IV = SLJ), BMI is inversely associated with SLJ (c = −0.019, *p* < 0.01), explaining about 10% of the total variance of SLJ (R^2^ = 9.92%). Moreover, an increase in PAL (Figure 1) results in better performance in SLJ (b = 0.091, *p* < 0.01) with higher variance across lower-limb strength (R^2^ = 17.16%). The same applies to Model 2 (Figure 2), where MBT is the IV: higher BMI improves motor performance in MBT (c = 0.087, *p* < 0.01), and higher self-reported PA enhances results in MBT (b = 0.250, *p* < 0.01). However, the addition of the mediation variable allowed for a slight understanding of the variation in upper-limb strength (ΔR^2^ = 0.0262). The third mediation model (Figure 3) assumes 10 × 5 as the IV. BMI negatively affects the 10 × 5 shuttle run (c = 0.248, *p* < 0.01), while children who are more physically active demonstrate a significant reduction in time spent performing the test (b = −0.837, *p* < 0.01), explaining more than 10.72% of the total variance.

In Figure 1, Figure 2 and Figure 3, c = total effect of independent variable on dependent variable; c’ = direct effect of independent variable on dependent variable adjusted per mediation variable; a = effect of independent variable on mediation variable; b = effect of mediation variable on dependent variable controlled per independent variable; a × b = indirect effects of independent variable on mediation variable, and mediation variable on dependent variable.

**Figure 4:**
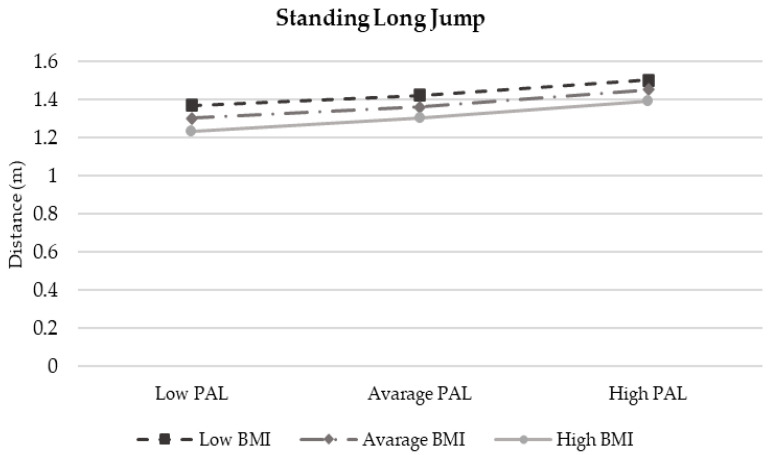
Moderation Model 1.

**Figure 5:**
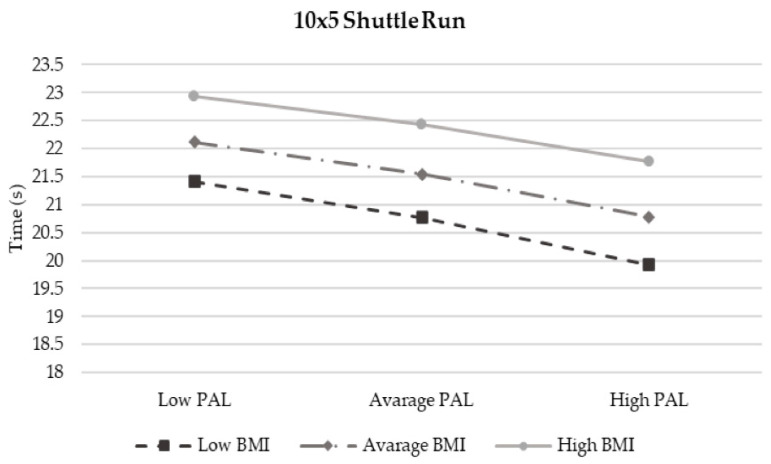
Moderation Model 3.

**Figure 6:**
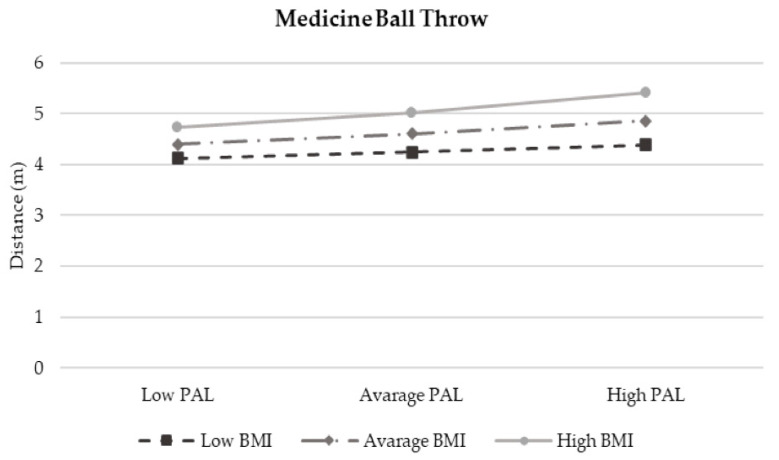
Moderation Model 2.

The moderation analysis according to Preacher and Hayes’ method is reported in Table 6. Despite the interaction term between BMI and PAL accounting for a certain proportion of the children’s physical fitness components (R2 Model 1 = 16.94%; R2 Model 2 = 12.84% and R2 Model 3 = 10.64%), these results are non-significant. Interaction plots (Figure 4 and Figure 5 show an enhancing effect whereby, as PAL increased and BMI decreased, lower-limb strength and agility improved. In contrast, both the increase in BMI and PAL led to better performance in MBT (Figure 6). All results, even if non-significant, confirm that an increase in PAL contributes to enhancing each component of physical fitness.

## 4. Discussion

The present study provides (R1) an analysis of physical fitness components and levels of physical activity in secondary schoolchildren according to gender and BMI cutoff. Results highlight that lower BMI is positively associated with higher lower-limb explosive strength, aerobic endurance, and levels of physical activity in both boys and girls, while upper-limb strength is directly associated with an increase in BMI. However, the findings reveal significant simple main effects for all variables according to BMI cutoff and gender (except for PAL), and a significant interaction effect for PAL.

The results of the present study are consistent with other studies conducted in Apulia as part of the ROMA Project, whose main findings revealed that increased BMI is negatively associated with motor performance [49,58].

Recent findings in the same field revealed that obese children showed better motor performance and explosive power compared to normal-weight children, while lower body weight was negatively associated with musculoskeletal strength [32]. As demonstrated in the present research, the study by Tsolakis et al. [26] highlighted that BMI was positively associated with upper-limb strength (assessed via the Hand Grip test), and negatively associated with cardiorespiratory fitness, lower-limb explosive strength, and trunk muscle endurance. Furthermore, sedentary lifestyles and reduced levels of physical activity led to an increase in overweight and obesity and a reduction in performance indicators of physical fitness components [59,60].

The second research question (R2) concerns the analysis of the mediating and moderating role of PAL between BMI and the development of physical fitness. In fact, although scientific evidence agrees that there is a negative association between BMI and physical fitness, the nature of this association remains to be clarified when considering the mediating and/or moderating effects of daily physical activity practice.

The results highlighted that PAL partially mediates the association between BMI and skill-related physical fitness. Data analyses revealed an inverse association between BMI, SLJ, and 10 × 5 and a positive correlation between BMI and MBT, but these models are mediated by daily practice of physical activity.

The findings in this paper are similar to those obtained in a previous study conducted in Apulia, but in just a single province: the results showed, in fact, a total mediating effect between BMI and the different components of physical fitness [49].

Findings in the literature confirm that higher levels of physical activity and physical fitness levels lead to a possible reduction in genetic predisposition to being overweight or obese during adulthood [61]. Moreover, studies reveal that structured physical activity interventions can improve body composition in children with overweight or obesity, regardless of age [62]. According to Wisnieski et al. [63], the development of aerobic capacity and the functionality of the cardiovascular system is a mediating factor between MVPA and body mass index (BMI). Furthermore, cross-sectional studies have already demonstrated that the incidence of overweight and obesity is strictly related to lower levels of physical activity [64,65]

A recent study [66] highlighted the importance of cardiorespiratory fitness as a full mediator between motor competence and daily physical activity in both boys and girls. Moreover, cardiorespiratory fitness serves as a complete mediator between obesity and PAL [67].

Findings from the present study align with other research in the same field, which indicates that the negative association between BMI, PAL, and physical fitness is closely related to the significant decline in MVPA in the 3-to-18-year age groups [68].

The obtained results gain significance when considering that higher speed–agility and muscle strength are also linked to other domains in children and adolescents, beyond the physical one. In fact, these physical fitness components play a mediating role in the negative association between excessive body weight and academic performance in children [69].

In addition, the moderation analysis showed no significant results. This may be due to inaccuracies in self-reporting physical activity levels by children, as reported by other studies [70,71]. These results also suggest a further reflection of the intensity of physical activity. According to Sardinha et al. [72], energy expenditure during vigorous physical activity was inversely associated with total and abdominal fat mass indices in obese children, independent of cardiorespiratory fitness levels. Moreover, recent findings highlighted the importance of balancing volume and intensity during PA to achieve different goals: increased volume is linked to improved bone-mineral content in children, and reduced fat mass in males, while intensity is associated with lean mass and fatness in both males and females [73].

In summary, although the results emphasize the negative association between increased BMI and motor performance, the scientific literature on the subject seems to attribute a significant role (e.g., direct or indirect effect) to physical activity practice.

### 4.1. Methodological and Didactic Implications for Physical Education Teachers

The results of the present study should be interesting and add to a better understanding of PE teachers’ behavior in the school setting, where the teacher is primarily responsible for enabling mediation functions among the students, the motor tasks, and the environment in which the motor experiences occur [74,75]. Furthermore, these findings assign significant value to the choice and the teachers’ reflection on the content and the organizational modalities to increase the levels of physical activity and the time of motor engagement during practice [76].

A thorough examination of teaching methodologies and organizational strategies in physical education is needed to enhance motor engagement during physical education classes and lessons. In fact, there is a positive association between time spent in moderate to vigorous physical activity and extended opportunities for motor skill learning [75].

Another study evaluated the effects of an experimental intervention protocol based on the learning of motor skills in a sample of overweight and obese children aged 6–7 years concerning the children’s routines at home, self-perception, body mass index, motor development, and the time spent in physical activity during physical education lessons [77]. The findings showed a decrease in television viewing and total screen time, and enhanced perceived motor competence, participation in physical activity, and motor skills, as well as a decrease in BMI [77]. In addition, the study by Ługowska et al. (2023) showed that an increase in PA at school (10 h per week) effectively improved physical fitness levels assessed using standing long jump, shuttle run, and core strength in adolescents aged 11–12 years old [78].

Further opportunities to promote and enhance time for motor engagement in school might include family involvement [79,80], as well as the proposal of multi-component projects at school [81,82] and experimental interventions aimed at developing PE teachers’ training courses regarding variables that may lead to an increase or decrease in PAL, such as self-perception, self-perceived competence, enjoyment, and motivation, as suggested in other studies [83,84,85,86,87].

### 4.2. Study Limitations and Future Research

This study highlights significant differences based on gender and BMI cutoff groups, as well as significant indirect effects of PAL in the association between BMI and physical fitness, suggesting a foundation for future scientific research. However, some limitations emerged and should be adequately considered. First, although the authors used cluster sampling to recruit the sample, the cutoff groups are quite unequal, making it difficult to generalize the results. Despite the authors applying Gabriel’s procedure for conducting post hoc analysis, the presence of unequal group sizes may still lead to higher variability that could influence the robustness of the findings. Future studies should use a stratified sampling approach.

Additionally, in this study, the authors used Cole’s classification [44] for normal weight, overweight, and obesity. Subsequent studies could, for example, classify children through percentile z-scores or the addition of other assessment tools (e.g., waist circumference, plicometry, etc.). Regarding the assessment methods, despite the authors proposing a validated questionnaire for assessing self-reported physical activity, the instrument may still introduce recall bias or inaccuracies, particularly in children, who may struggle to report their activity levels accurately.

A further limitation is the lack of additional covariates, in this specific case age. The authors’ objective was, in fact, to create a possible explanatory model applicable to secondary schools in Italy. Future research will focus on defining specific age-dependent models of mediation and moderation, as well as the assessment of the mediating and/or moderating effects of different activities conducted during physical education classes on physical fitness. Moreover, to validate these preliminary findings, future research should employ longitudinal designs to examine differences among various age groups and genders, including children with disabilities and/or physical/cognitive impairments. Finally, it would be interesting to conduct and assess the effectiveness of experimental interventions in schools for enhancing physical fitness and promoting healthy behaviors in adolescents.

## 5. Conclusions

The present study suggests the important role of physical activity in mediating the association between BMI and physical fitness in Italian children. Despite the non-significant moderation effect, the findings indicate that higher levels of physical activity result in better motor performance in children with higher BMI compared to those with average and low BMI. The developed mediation models confirm the effect of BMI on physical fitness components via PAL, suggesting that BMI affects PAL, which in turn influences motor performance. Instead, the results of moderation models show no significant conditional effect, suggesting that the influence of BMI on physical fitness depends on the PAL (as a moderator). However, these data should be properly interpreted in light of the mentioned limitations of this study. Future research should also be performed using different research designs and a deeper analysis of sociocultural determinants of physical activity for the generalization of the results.

## Figures and Tables

**Table 1:** Sample’s anthropometric profile.

**Male *n* = 196**						
	**Nw = 114**		**Ow = 58**		**Ob = 24**	
	**M ± SD**	**Min–Max**	**M ± SD**	**Min–Max**	**M ± SD**	**Min–Max**
Age	12.27 ± 0.87	11–14	12.05 ± 0.85	11–14	12.04 ± 0.95	11–14
Height (m)	1.53 ± 0.09	1.29–1.79	1.57 ± 0.08	1.40–1.77	1.57 ± 0.96	1.38–1.78
Weight (Kg)	43.58 ± 8.93	24.00–71.80	57.38 ± 7.48	44.00–71.00	71.00 ± 9.42	54.00–92.00
BMI (kg/m^2^)	18.17 ± 2.01	13.47–21.86	22.98 ± 1.39	20.50–26.10	28.40 ± 2.35	25.22–35.41
SLJ (m)	1.56 ± 0.25	0.70–2.17	1.38 ± 0.22	1.00–2.20	1.30 ± 0.28	0.87–1.90
MBT Kg2 (m)	4.93 ± 1.16	2.50–8.24	5.14 ± 1.18	3.35–8.13	5.16 ± 1.24	2.90–8.10
10 × 5 (s)	18.52 ± 3.57	11.32–26.68	20.16 ± 3.83	13.83–32.73	21.29 ± 3.80	13.73–26.39
PAL	2.91 ± 0.75	1.16–4.37	2.62 ± 0.78	1.39–4.58	2.05 ± 0.41	1.57–2.85
**Female *n* = 191**						
	**Nw = 126**		**Ow = 53**		**Ob = 12**	
	**M ± SD**	**Min–Max**	**M ± SD**	**Min–Max**	**M ± SD**	**Min–Max**
Age	12.37 ± 0.96	11–14	12.23 ± 0.82	11–14	12.17 ± 1.19	11–14
Height (m)	1.53 ± 0.08	1.28–1.77	1.55 ± 0.08	1.31–1.73	1.59 ± 0.04	1.52–1.67
Weight (Kg)	44.20 ± 8.37	25.00–65.20	57.98 ± 8.60	40.00–76.90	73.67 ± 5.67	65.00–85.00
BMI (kg/m^2^)	18.46 ± 2.21	13.14–22.70	23.87 ± 1.60	20.77–27.34	29.13 ± 2.40	25.65–35.84
SLJ (m)	1.32 ± 0.21	0.67–2.05	1.21 ± 0.17	0.80–1.51	1.11 ± 0.19	0.86–1.55
MBT Kg2 (m)	4.13 ± 0.91	2.30–7.20	4.47 ± 0.86	2.75–7.00	5.30 ± 1.45	3.20–8.40
10 × 5 (s)	20.48 ± 4.01	12.82–31.50	22.54 ± 4.17	14.78–32.84	26.22 ± 2.64	19.18–29.39
PAL	2.47 ± 0.74	1.00–4.57	2.26 ± 0.61	1.32–4.14	2.35 ± 0.64	1.43–3.24

**Table 2:** Effect of gender and BMI cutoff on physical fitness and PAL.

	Gender				CutOff				Gender × Cutoff			
	**df**	**F**	***p***	**η^2^**	**df**	**F**	***p***	**η^2^**	**df**	**F**	***p***	**η^2^**
SLJ	1	38.604	<0.01	0.082	2	24.824	<0.01	0.105	2	1.058	0.348	0.004
MBT	1	8.525	<0.01	0.021	2	7.363	<0.01	0.036	2	2.761	0.065	0.013
10 × 5	1	32.736	<0.01	0.071	2	22.234	<0.01	0.096	2	2.101	0.124	0.009
PAL	1	2.251	0.113	0.008	2	8.229	<0.01	0.050	2	3.452	<0.01	0.021

**Table 3:** Post hoc analysis.

	Gender					Groups				
SLJ	Female < male; *p* < 0.01; *d* = 0.824					Nw > Ow; *p* < 0.01; *d* = 0.530				
						Nw > Ob; *p* < 0.01; *d* = 0.774				
						Ow > Ob; *p =* 0.130; *d* = 0.244				
MBT	Female < male; *p* < 0.01; *d* = 0.622					Nw < Ow; *p* = 0.064; *d* = 0.277				
						Nw < Ob; *p* < 0.01; *d* = 0.620				
						Ow < Ob; *p =* 0.118; *d* = 0.343				
10 × 5	Female > male; *p* < 0.01; *d* = 0.512					Nw < Ow; *p* < 0.01; *d* = 0.436				
						Nw < Ob; *p* < 0.01; *d* = 0.843				
						Ow < Ob; *p* < 0.01; *d* = 0.407				
PAL	Female < male; *p* = 0.113; *d* = 0.428					Nw > Ow; *p* < 0.05; *d* = 0.302				
						Nw > Ob; *p* < 0.01; *d* = 0.720				
						Ow > Ob; *p =* 0.238; *d* = 0.418				
	Male					Female				
	F	*p*	ηp^2^	Groups		F	*p*	ηp^2^	Groups	
SLJ	16.771	<0.01	0.148	Nw > Ow	<0.01	9.150	<0.01	0.089	Nw > Ow	<0.01
				Nw > Ob	<0.01				Nw > Ob	<0.01
				Ow > Ob	0.380				Ow > Ob	0.299
										
MBT	0.786	0.457	0.008	Nw < Ow	0.520	9.827	<0.01	0.095	Nw < Ow	0.067
				Nw < Ob	0.665				Nw < Ob	<0.01
				Ow < Ob	0.997				Ow < Ob	0.017
										
10 × 5	7.682	<0.01	0.074	Nw < Ow	0.017	14.231	<0.01	0.131	Nw < Ow	<0.01
				Nw < Ob	<0.01				Nw < Ob	<0.01
				Ow < Ob	0.417				Ow < Ob	0.012
										
PAL	11.850	<0.01	0.129	Nw > Ow	0.069	1.456	0.236	0.019	Nw > Ow	0.214
				Nw > Ob	<0.01				Nw > Ob	0.837
				Ow > Ob	<0.01				Ow < Ob	0.917

**Table 4:** Correlation between variables.

	SLJ	MBT	10 × 5	PAL
BMI	−0.257 **	0.289 **	0.224 **	−0.228 **
SLJ	/	0.429 **	−0.356 **	0.334 **
MBT	/	/	−0.090	0.089
10 × 5	/	/	/	−0.233 **

** = *p* < 0.01.

**Table 5:** Analysis of indirect effect of Models 1, 2, and 3.

	Direct Effect					Indirect Effect					
			Bootstrap					Bootstrap			ΔR^2^
	R^2^	Value	LLCI	ULCI	*p*	R^2^_1_	Value	LLCI	ULCI	*p*	
Model 1	0.099	−0.016	−0.026	−0.013	<0.01	0.171	−0.003	−0.006	−0.002	<0.01	0.0724
Model 2	0.091	0.098	0.057	0.117	<0.01	0.117	−0.011	−0.206	−0.004	<0.01	0.0262
Model 3	0.076	0.211	0.153	0.342	<0.01	0.107	0.037	0.013	0.068	<0.01	0.0303

LLCI = lower limit of confidence interval; ULCI = upper limit of confidence interval.

**Table 6:** Moderation analysis and interaction effect between BMI and PAL (INT) in Models 1, 2, and 3.

Moderation Analysis						
			Bootstrap			
		Value	LLCI	ULCI	*p*	R^2^
Model 1	BMI	−0.023	−0.048	0.0023	0.074	16.94
	PAL	0.032	−0.169	0.2347	0.752	
	INT	0.003	−0.007	0.0127	0.568	
Model 2	BMI	0.017	−0.100	0.1355	0.770	12.84
	PAL	−0.389	−1.339	0.561	0.421	
	INT	0.033	−0.013	0.079	0.161	
Model 3	BMI	0.146	−0.226	0.517	0.441	10.64
	PAL	−1.374	−4.364	1.617	0.367	
	INT	0.027	−0.119	0.0173	0.719	

## Data Availability

The data presented in this study are available on request from the corresponding author. Data are unavailable due to privacy or ethical restrictions.

## Abbreviations

The following abbreviations are used in this manuscript:

BMI	Body mass index
SLJ	Standing long jump
MBT	Medicine ball throw
10 × 5	Shuttle run 10 × 5
PAL	Level of physical activity
WHO	World Health Organization
MET	Metabolic equivalent task
